# Prognostic Value of Right Ventricular 3D Speckle-Tracking Strain and Ejection Fraction in Patients With HFpEF

**DOI:** 10.3389/fcvm.2021.694365

**Published:** 2021-06-30

**Authors:** Yuanli Meng, Shuangshuang Zhu, Yuji Xie, Yanting Zhang, Mingzhu Qian, Lang Gao, Meng Li, Yixia Lin, Wenqian Wu, Jing Wang, Yali Yang, Qing Lv, Li Zhang, Yuman Li, Mingxing Xie

**Affiliations:** ^1^Department of Ultrasound Medicine, Union Hospital, Tongji Medical College, Huazhong University of Science and Technology, Wuhan, China; ^2^Clinical Research Center for Medical Imaging in Hubei Province, Wuhan, China; ^3^Hubei Province Key Laboratory of Molecular Imaging, Wuhan, China; ^4^Department of Cardiology, Sun Yat-sen Memorial Hospital, Sun Yat-sen University, Guangzhou, China; ^5^Shenzhen Huazhong University of Science and Technology Research Institute, Shenzhen, China; ^6^Wuhan National Laboratory for Optoelectronics, Tongji Medical College, Huazhong University of Science and Technology, Wuhan, China

**Keywords:** heart failure with preserved ejection fraction, speckle tracking echocardiography, right ventricular, strain, prognosis

## Abstract

**Background:** Right ventricular longitudinal strain of free wall (RV FWLS) assessed by two-dimensional speckle-tracking echocardiography (2D-STE) is recognized as an independent predictor of poor prognosis in patients with heart failure with preserved ejection fraction (HFpEF). However, the prognostic implications of three-dimensional STE (3D-STE) parameters in patients with HFpEF have not been well-established. The purpose of our study was to determine whether 3D-STE parameters were the more powerful predictors of poor outcomes in HFpEF patients compared with 2D-STE indices.

**Methods:** Eighty-one consecutive patients with HFpEF were studied by 2D-STE and 3D-STE. RV volumes, ejection fraction (EF) and 3D-RVFWLS were measured by 3D-STE. 2D-RVFWLS was determined by 2D-STE. Patients were followed for the primary end point of heart failure (HF)-related hospitalization and death for HF.

**Results:** After a median follow-up period of 17 months, 39 (48%) patients reached the end point of cardiovascular events. Compared with HFpEF patients without end-point events, those with end-point events had lower RVEF and 3D-RVFWLS (*P* < 0.05). Separate multivariate Cox regression analyses revealed that 3D-RVFWLS (HR 5.73; 95% CI 2.77–11.85; *P* < 0.001), RVEF (HR 3.47; 95% CI 1.47–8.21; *P* = 0.005), and 2D-RVFWLS (HR 3.17; 95% CI 1.54–6.53; *P* = 0.002) were independent predictors of adverse outcomes. The models with 3D-RVFWLS (AIC = 246, C-index = 0.75) and RVEF (AIC = 247, C-index = 0.76) had similar predictive performance for future clinical events as with 2D-RVFWLS (AIC = 248, C-index = 0.74).

**Conclusions:** 3D-STE parameters are powerful predictors of poor outcomes, providing a similar predictive value as 2D-STE indices in patients with HFpEF. These findings support the potential of RV 3D-STE to identify HFpEF patients at higher risk for adverse cardiac events.

## Introduction

Heart failure with preserved ejection fraction (HFpEF) accounts for half of heart failure (HF) patients and has been established as a major cause of cardiovascular mortality (1). Prior studies have demonstrated that right ventricular (RV) dysfunction is a powerful predictor of mortality and morbidity in patients with HFpEF (2–4). Accurate evaluation of RV function is highly desired for the treatment of these patients. Echocardiography, as a non-invasive and widely available technique, has been demonstrated to be a good first-line tool for the evaluation of cardiac function. However, accurate assessment of RV function using conventional echocardiography remains challenging owing to the RV complex structure. The conventional RV function echocardiographic parameters recommended by guidelines, which include RV fractional area change (RVFAC), tricuspid annular myocardial tissue Doppler velocities (S′), and tricuspid annular plane systolic excursion (TAPSE), have its own limitations (5, 6).

Recently, two-dimensional (2D) speckle tracking echocardiography (STE) has been considered to be a sensitive, reliable quantitative technique for RV function assessment (7–9). Furthermore, growing evidence suggests that RV longitudinal strain of free wall (RV FWLS) derived from 2D-STE provides incremental prognostic information over standard RV parameters in varying clinical settings, including patients with pulmonary arterial hypertension, HF with reduced ejection fraction, or coronavirus disease-2019 (10–12). However, 2D-STE analysis has some primary limitations, such as foreshortened views, geometric modeling, and out-of-plane motion of the speckles. Moreover, 2D-STE evaluation is only available in the apical four-chamber view, precluding the assessment of RV outflow portions. Three-dimensional (3D) STE has been introduced as a novel technique that allows an accurate and comprehensive assessment of ventricular function due to overcoming the aforementioned limitations (13, 14). To our knowledge, the prognostic value of 3D-STE in patients with HFpEF has not been described.

Accordingly, the aims of our study were to assess RV systolic function in patients with HFpEF using 2D- and 3D-STE and determine whether 3D-STE parameters provide similar predictive values as 2D-STE indices.

## Materials and Methods

### Study Population

A total of 93 consecutive patients with HFpEF referred to Wuhan Union hospital between March 2016 and December 2018 were enrolled in our study. HFpEF was defined according to the following diagnostic criteria (15): (1) a primary diagnosis of HF as defined by Framingham criteria, (2) left ventricular ejection fraction (LVEF) ≥ 50%, (3) B-type natriuretic peptide (BNP) ≥ 35 pg/ml or N-terminal pro-BNP ≥ 125 pg/ml, (4) left atrial volume index (LAVI) ≥ 34 ml/m^2^, or a left ventricular mass index (LVMI) ≥115 g/m^2^ for males and ≥95 g/m^2^ for females, and/or (5) left ventricular (LV) diastolic dysfunction. A comprehensive approach was used to diagnose the LV diastolic dysfunction according to the recommendations of the American Society of Echocardiography and the European Association of Cardiovascular Imaging. Patients with septal e′ <7 cm/s, lateral e′ < 10 cm/s, average E/e′ ratio > 14, LA volume index > 34 ml/m^2^, and peak TR velocity > 2.8 m/s are considered to have diastolic dysfunction (16). Exclusion criteria were significant left-sided valve diseases, known cardiomyopathies, congenital heart disease, pericardial disease, severe chronic obstructive or interstitial pulmonary disease, mitral valve replacement, organic valvular diseases, acute coronary syndrome, or hemodynamic instability. Of these patients, 12 patients with insufficient image quality for strain analysis were excluded. The remaining 81 patients with HFpEF were included in our final analysis. This study was approved by the Ethics Committee of Tongji Medical College, Huazhong University of Science and Technology. All participants provided written informed consent.

### Echocardiography

All subjects underwent comprehensive transthoracic 2D, Doppler, and 3D echocardiographic examination using commercially available systems (Philips iE33; Philips Medical Systems, Andover, MA, USA). All participants were placed in the left lateral position, and the electrocardiography was recorded simultaneously. During end-expiratory breath holding, 2D and 3D images were obtained. All of the echocardiographic parameters were measured three times, and the mean value was used for the statistical analysis.

### Conventional Echocardiography

Cardiac chamber sizes, and LV and RV function were measured based on the guidelines of the American Society of Echocardiography (6). Left atrial (LA) volume was measured from the apical four- and two-chamber views. LV mass was calculated from the parasternal view on the basis of Devereux's formula. LVEF was obtained by biplane Simpson's method. LV diastolic function was assessed using the ratio of early transmitral flow velocity (E) to early diastolic septal tissue velocity (e′) in the apical four-chamber view. RV basal and mid transverse diameters, and longitudinal dimension, were determined from the apical four-chamber view. RV end-diastolic and end-systolic areas were measured from the apical four-chamber view to calculate RVFAC. RVFAC was calculated as [(RV end-diastolic area—RV end-systolic area)/RV end-diastolic area] × 100%. TAPSE was obtained using M-mode echocardiography of the lateral annulus. S′ was assessed using tissue Doppler imaging from the apical four-chamber view. Pulmonary artery systolic pressure (PASP) was estimated from peak tricuspid regurgitation jet velocity, using the simplified Bernoulli equation and combining this value with an estimate of the right atrial pressure. Right atrial pressure was estimated from the inferior vena cava diameter and its respiratory changes.

### 2D-STE Analysis

2D grayscale images were obtained for the subjects using the RV-focused apical four-chamber view at frame rates of 50–90 frames/s. All of the images were digitally stored for offline analysis using commercially available software (2D Cardiac Performance Analysis 1.2; TOMTEC Imaging Systems GmbH, Unterschleissheim, Germany). The RV endocardial border was manually traced in the end-systolic frame when the endocardial border was the clearest during the cardiac cycle. The region of interest (ROI) in each image was automatically generated. The software then automatically tracked the speckle patterns in the myocardium frame by frame. The position of ROI and its width were adjusted manually when the speckle tracking appeared to be poor. Finally, the software automatically generated the RV longitudinal strain curves, in which the peak longitudinal strain of each segment was measured. 2D RVFWLS (2D-RVFWLS) was calculated as the average value of the basal, middle, and apical segments of the RV free wall.

### 3D-STE Analysis

**Figure 1:**
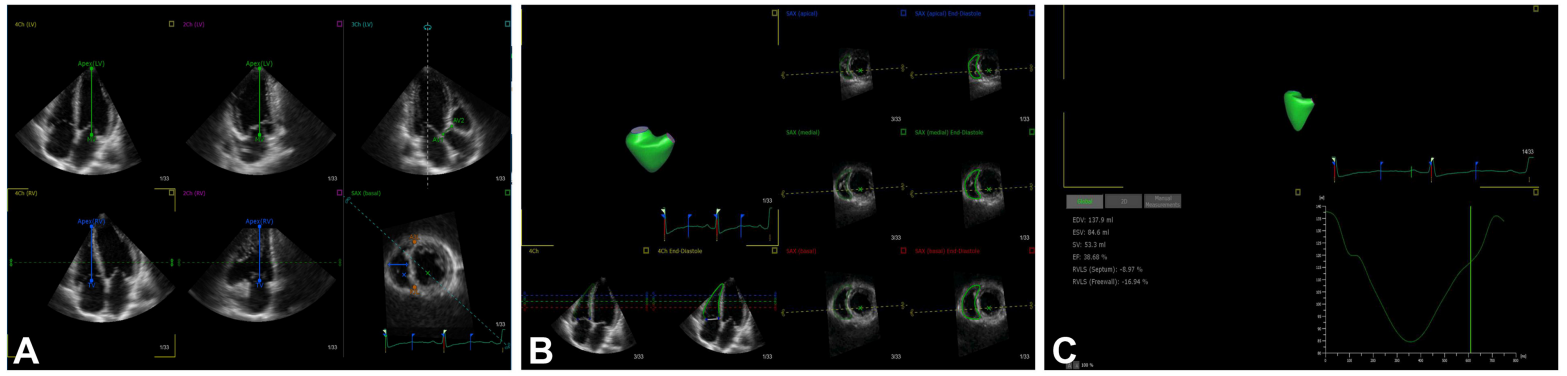
3D-STE offline analysis. **(A)** Setting reference points; **(B)** RV endocardial border identification and tracking; **(C)** longitudinal strain of RV free wall and RV volume curve were automatically generated.

3D echocardiographic acquisitions were obtained using the RV-focused apical four-chamber view in full-volume mode with volume rates of 20–35 volumes/s. The 3D full-volume data sets combining four subvolumes were analyzed using 3D speckle tracking software (4D RV Analysis, version 2.0; TOMTEC Imaging Systems GmbH, Unterschleissheim, Germany). The selected 3D data sets were displayed as multi-planar reconstruction images containing three standard long-axis views of the left ventricle (apical four-chamber, apical two-chamber, and apical three-chamber), two long-axis views of the right ventricle (apical four-chamber and apical two-chamber), and a RV short-axis view. In the apical four- and two-chamber views, the largest apical long-axis dimensions were set by the point of the LV apex and the center of the mitral annular line. In the apical three-chamber view, the operator set the landmarks corresponding to the aortic annulus diameter (AV1–AV2, Figure 1A). The point of the RV apex and the center of the tricuspid annular line were obtained in the RV apical four-chamber and coronal views. In the RV short-axis view, the distance between anterior and posterior junctions of the RV free wall with the interventricular septum and the distance of the septum-to-RV free wall were set (Figure 1A). The software automatically tracked the RV contours throughout the entire cardiac cycle, which was manually modified if necessary (Figure 1B). The software finally generated an RV volume curve, RV end-diastolic volume (RVEDV), RV end-systolic volume (RVESV), RV ejection fraction (RVEF), and 3D RVFWLS (3D-RVFWLS) (Figure 1C). 3D-RVFWLS was calculated as the average value of three segments of the RV free wall.

### Follow-Up

Patients were prospectively followed until December 2020. Follow-up information was obtained by clinical visits or by telephone contacts with the patients or their relatives. The end points consisted of death for HF or rehospitalization due to worsening of HF.

### Statistical Analysis

Continuous numeric variables are reported as mean ± SD or median (interquartile range) and were compared using a two-sample Student's *t*-test for normally distributed data or the Mann–Whitney *U*-test for non-normally distributed data. The Kolmogorov–Smirnov-test was used to determine the data distribution. Categorical variables are reported as numerical values and percentage and were compared using the Chi-square test or the Fisher exact-test. Correlation analysis was performed using the Pearson correlation coefficient. The receiver operator characteristic (ROC) curves were used to determine the optimal cutoff value of RV function indices for the detection of poor outcomes. Univariable and multivariable Cox proportional-hazard models were used to determine the predictors of unfavorable clinical outcomes. The following covariates were included in the univariate analyses: age, sex, blood pressure, heart rate, NYHA class, comorbidities, laboratory findings, and conventional LV and RV function, 2D-STE, and 3D-STE parameters. To avoid both the multi-collinearity among RV measurements and overfitting issues, a separate Cox proportional-hazard model including clinical variables and one of the RV function parameters (RVFAC, RVEF, 2D-RVFWLS, or 3D-RVFWLS) was used to determine the independent predictors of adverse outcomes. Model performance was assessed using the Akaike information criterion (AIC) and the C-index. Survival curves were obtained using the Kaplan–Meier method. The log-rank test was used to estimate the differences between the two groups. Statistical analyses were performed using SPSS for Window version 17.0 (SPSS, Inc., Chicago, IL, USA), MedCalc software (Version 19.0.4, Ostend, Belgium), and R software (version 3.6.3, R Foundation for Statistical Computing, Vienna, Austria). Two-sided *P*-values <0.05 were considered statistically significant.

Intra-observer variability and inter-observer variability were assessed in 20 randomly selected patients with HFpEF. Intra-observer variability was determined by having one observer remeasure after 2 months. Inter-observer variability was determined by a second observer who was blinded to the first observer's measurements. Intra-observer and inter-observer reproducibility were evaluated by means of intra-class correlation coefficient (ICC) and Bland–Altman analysis.

## Results

### Correlations of RVEF With STE and Conventional RV Function Parameters

**Figure 2:**
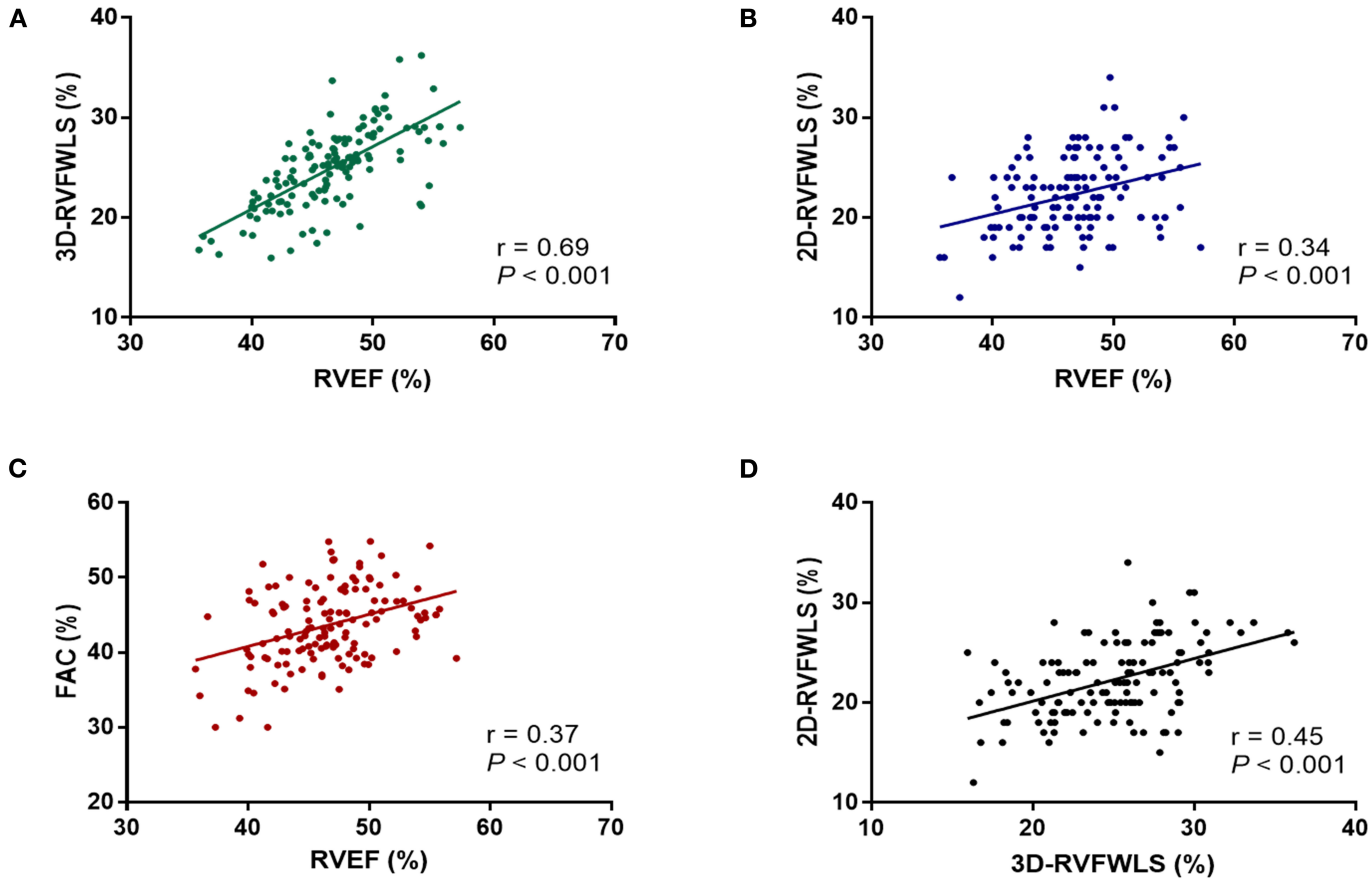
Correlations of RVEF with 2D- and 3D-STE, and conventional echocardiographic parameters. RVEF was correlated with 3D-RVFWLS **(A)**, 2D-RVFWLS **(B)**, and RVFAC **(C)**; 3D-RVFWLS was also weakly associated with 2D-RVFWLS **(D)**. 2D-RVFWLS, two-dimensional right ventricular free wall longitudinal strain; 3D-RVFWLS, three-dimensional right ventricular free wall longitudinal strain; RVEF, right ventricular ejection fraction; RVFAC, right ventricular fractional area change. 2D- and 3D-RVFWLS values are absolute values.

RVEF was moderately correlated with 3D-RVFWLS (*r* = 0.69; *P* < 0.001) and weakly with 2D-RVFWLS (*r* = 0.34; *P* < 0.001) and RVFAC (*r* = 0.37; *P* < 0.001) (Figures 2A–C). Furthermore, 3D-RVFWLS correlated better than 2D-RVFWLS and RVFAC with RVEF (0.69 vs. 0.34; *P* = 0.001; 0.69 vs. 0.37; *P* = 0.002; respectively). In addition, a weak correlation between 3D-RVFWLS and 2D-RVFWLS was observed in our study (*r* = 0.45; *P* < 0.001) (Figure 2D). 3D-RVFWLS, RVEF, and 2D-RVFWLS were also weakly associated with PASP (*r* = −0.31; *P* = 0.001; *r* = −0.28; *P* = 0.002; *r* = −0.40; *P* < 0.001; respectively). LVEF was not correlated with 3D-RVFWLS, 2D-RVFWLS, and RVEF (all *P* > 0.05).

### Clinical Outcomes

The median follow-up time was 17 (11–36) months. A total of 39 (48%) patients reached the end point of events: 6 (15%) patients died, and 33 (85%) were hospitalized for worsening HF. The clinical and echocardiographic characteristics in HFpEF patients with/without end-point events are depicted in Table 1. Compared with patients without end-point events, those with events had higher systolic blood pressure (142 ± 25 mmHg vs. 131 ± 19 mmHg, *P* = 0.022) and BNP level [84 (54, 190) pg/ml vs. 73 (46, 123) pg/ml, *P* < 0.001]. Regarding echocardiographic data, compared with event-free patients, those with events exhibited a higher E/e′ ratio (17 ± 8 vs. 13 ± 4, *P* = 0.005), larger RV volumes (RVEDVi: 59 ± 29 ml/m^2^ vs. 47 ± 17 ml/m^2^, *P* = 0.037; RVESVi: 33 ± 18 ml/m^2^ vs. 25 ± 8 ml/m^2^, *P* = 0.019), and lower RVEF (44 ± 5% vs. 47 ± 4%, *P* = 0.005) and 3D-RVFWLS (−23 ± 4% vs. −25 ± 5%, *P* = 0.001) whereas 2D-RVFWLS and standard RV function parameters were similar between two subgroups.

**Table 1:** Basic and echocardiographic characteristics of HFpEF patients without or with end-point event.

**Variables**	**Without end point (*n* = 42)**	**With end point (*n* = 39)**	***P-*value**
**Basic characteristics**			
Age (y)	61 ± 12	63 ± 13	0.570
Male gender (%)	27 (64%)	26 (67%)	0.825
Heart rate (b.p.m)	71 ± 11	74 ± 12	0.296
SBP (mmHg)	131 ± 19	142 ± 25^*^	0.022
DBP (mmHg)	83 ± 15	83 ± 15	0.851
BMI (kg/m^2^)	26 ± 4	25 ± 3	0.284
Smoke (%)	24 (57%)	21 (54%)	0.769
Diabetes (%)	19 (45%)	17 (44%)	0.883
Hypertension (%)	21 (50%)	25 (62%)	0.200
AF (%)	4(10%)	6(15%)	0.429
New York Heart Association class			
I/II (%)	31 (74%)	29 (74%)	0.951
III/IV (%)	11 (26%)	10 (26%)	
BNP (pg/ml)	73 (46, 123)	84 (54, 190)^*^	<0.001
Creatinine (mg/dl)	87 (71, 152)	99 (77, 142)	0.360
**Echocardiographic characteristics**			
LVEF (%)	66 ± 5	64 ± 4	0.186
E/e′	13 ± 4	17 ± 8^*^	0.005
LVMI (g/m^2^)	101 ± 26	87 ± 26	0.082
LAVI (mL/m^2^)	44 ± 13	42 ± 9	0.437
RVD1 (mm)	33 ± 5	37 ± 10	0.070
RVD2 (mm)	30 ± 7	33 ± 10	0.139
RVD3 (mm)	69 ± 9	71 ± 10	0.388
PASP (mmHg)	30 ± 4	33 ± 7	0.440
TAPSE (mm)	20 ± 3	20 ± 3	0.640
RVFAC (%)	42 ± 4	41 ± 5	0.273
S′ (cm/s)	11 ± 2	12 ± 3	0.052
2D-RVFWLS (%)	−21 ± 3	−20 ± 4	0.109
RVEDVi (ml/m^2^)	47 ± 17	59 ± 29^*^	0.037
RVESVi (ml/m^2^)	25 ± 8	33 ± 18^*^	0.019
RVEF (%)	47 ± 4	44 ± 5^*^	0.005
3D-RVFWLS (%)	−25 ± 5	−23 ± 4^*^	0.001

*2D, two dimensional; 3D, three dimensional; AF, atrial fibrillation; BNP, B-type natriuretic peptide; DBP, diastolic blood pressure; SBP, systolic blood pressure; LVEF, left ventricular ejection fraction; LVMI, left ventricular mass index; LAVI, left atrial volume index; RVEF, right ventricular ejection fraction; RVD, right ventricular dimension; RVFWLS, right ventricular free wall longitudinal strain; RVFAC, right ventricular fractional area change; TAPSE, tricuspid annular plane systolic excursion; RVEDVI, right ventricular end-diastolic volume index; RVESVI, right ventricular end-systolic volume index; PASP, pulmonary artery systolic pressure*.

* *P < 0.05 compared with HFpEF patients without end-point event*.

3D-STE, 2D-STE, and hemodynamic and conventional RV function parameters were entered into a ROC analysis to evaluate the probability of adverse clinical outcomes.

**Figure 3:**
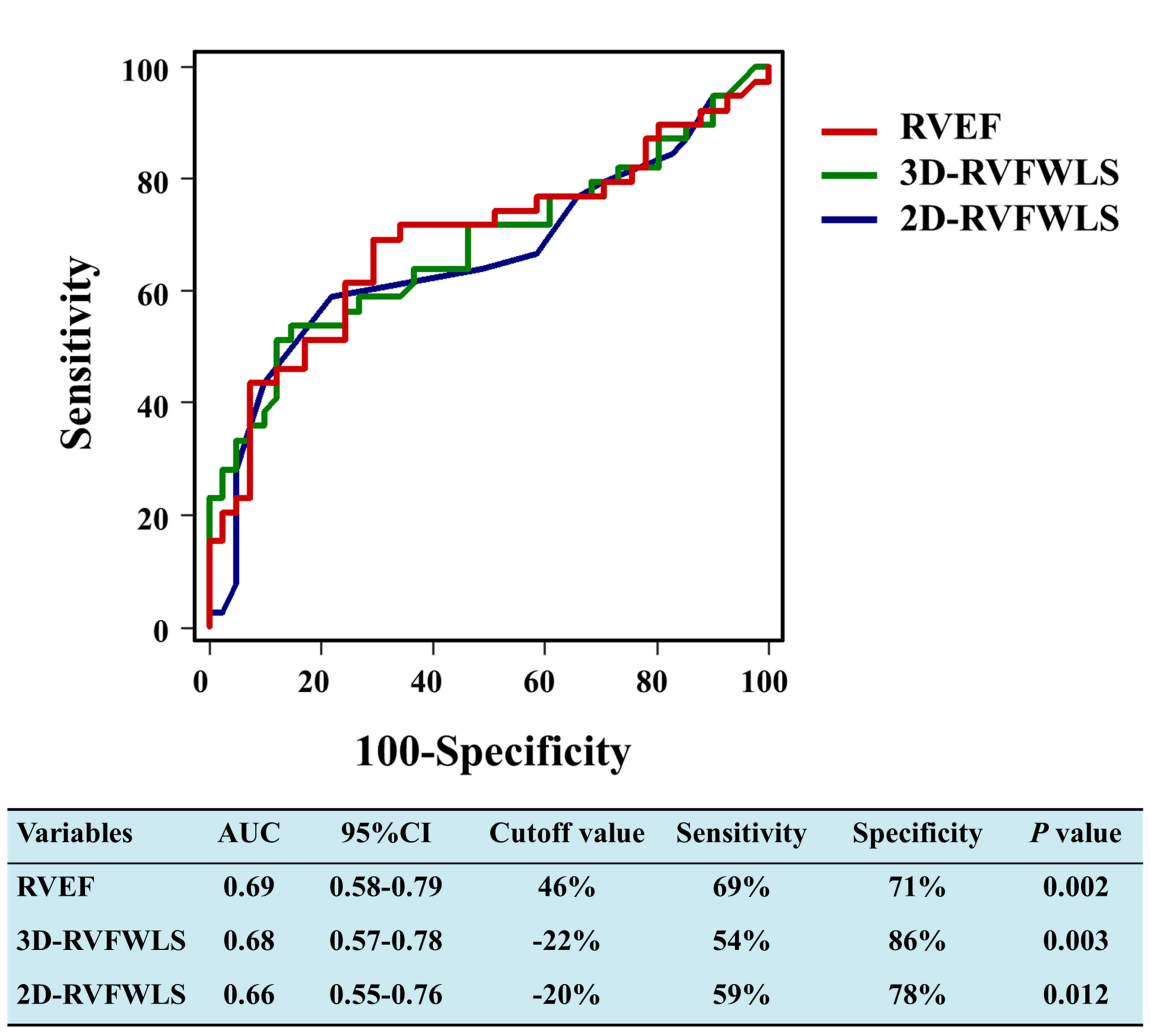
Receiver-operating characteristic curves of RVEF, 2D-RVFWLS, and 3D-RVFWLS for adverse clinical outcomes. RVEF, 2D-RVFWLS, and 3D-RVFWLS were associated with poor outcomes in patients with HFpEF. Other abbreviations are shown in Figure 2.

**Figure 4:**
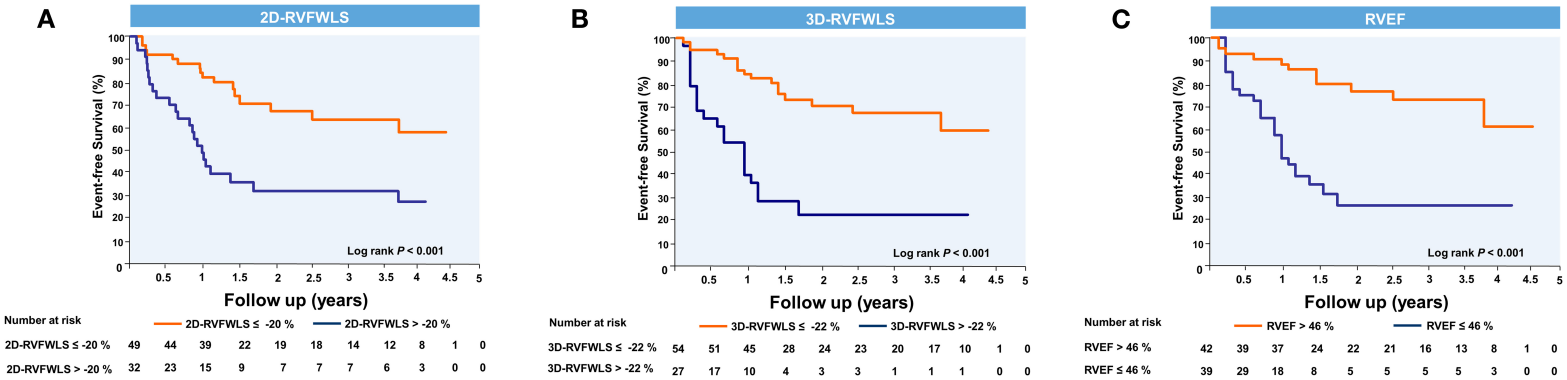
Kaplan–Meier event-free survival curves showing the association of right ventricular 2D- and 3D-STE parameters and RVEF with higher risk of adverse clinical events. Kaplan–Meier curves of event-free survival in patients stratified by the cutoff values of 2D-RVFWLS **(A)**, 3D-RVFWLS **(B)**, and RVEF **(C)**. Abbreviations as in Figure 2.

ROC analyses for unfavorable outcomes revealed that 3D-RVFWLS, 2D-RVFWLS, and RVEF were associated with adverse clinical events (Figure 3). Areas under the curve for 3D-RVFWLS, RVEF, and 2D-RVFWLS were 0.68, 0.69, and 0.66, respectively. We found that 3D-RVFWLS and RVEF have a similar diagnostic performance for detecting adverse outcomes as 2D-RVFWLS (0.68 vs. 0.66, *P* > 0.05; 0.69 vs. 0.66, *P* > 0.05). The best cutoff values of 3D-RVFWLS and RVEF for the detection of poor outcomes were −22% (sensitivity, 54%; specificity, 86%) and 46% (sensitivity, 69%; specificity, 71%), respectively. A 2D-RVFWLS cutoff value of −20% had a sensitivity of 59% and a specificity of 78% for identifying end-point events. Kaplan–Meier survival curves stratified by the cutoff values of 3D-RVFWLS, 2D-RVFWLS, and RVEF are presented in Figure 4. 3D-RVFWLS lower than 22%, 2D-RVFWLS lower than 20%, or RVEF lower than 46% was associated with adverse outcomes. They clearly revealed that event-free survival significantly declined with worsening RV longitudinal strain and RVEF.

In univariate Cox regression analyses, high SBP [HR 1.24; 95% confidence interval (CI): 1.05–1.48; *P* = 0.013], BNP level (HR 1.43; 95% CI: 1.12–1.84; *P* = 0.005), and E/e′ (HR 1.13; 95% CI: 1.07–1.20; *P* < 0.001) and decreased RVFAC (HR 0.92; 95% CI: 0.84–0.99; *P* = 0.038), RVEF (HR 0.73; 95% CI: 0.67–0.80; *P* < 0.001), 2D-RVFWLS (HR 1.12; 95% CI: 1.01–1.24; *P* = 0.035), and 3D-RVFWLS (HR 1.26; 95% CI: 1.14–1.41; *P* < 0.001) were predictors of poor clinical outcomes in patients with HFpEF (Table 2). In multivariate Cox analysis models, elevated BNP level continued to be of predictive value. Separate multivariate Cox regression analyses revealed that 3D-RVFWLS (HR 5.73; 95% CI: 2.77–11.85; *P* < 0.001), RVEF (HR 3.47; 95% CI 1.47–8.21; *P* = 0.005), and 2D-RVFWLS (HR 3.17; 95% CI 1.54–6.53; *P* = 0.002) were independent predictors of adverse outcomes (Table 3). The models with 3D-RVFWLS (AIC = 246, C-index = 0.75) and RVEF (AIC = 247, C-index = 0.76) had similar predictive performance for future clinical events as with 2D-RVFWLS (AIC = 248, C-index: 0.75 vs. 0.74, *P* = 0.74; 0.76 vs. 0.74, *P* = 0.91).

**Table 2:** Univariate analysis of predictors of poor outcomes in patients with heart failure and preserved ejection fraction.

**Variables**	**Univariate cox regression**	
	**HR (95% CI)**	***P***
Age (y)	1.01 (0.99–1.04)	0.456
Male	1.04 (0.53–2.02)	0.918
BMI (kg/m^2^)	0.98 (0.90–1.07)	0.66
SBP, per 10 mmHg	1.24 (1.05–1.48)	0.013
SDP, per 10 mmHg	1.03 (1.02–1.05)	0.82
Heart rate (b.p.m)	1.03 (0.99–1.06)	0.079
NYHA class III	1.19 (0.72–1.97)	0.494
Diabetes	1.04 (0.55–1.96)	0.908
Smoke	0.89 (0.47–1.67)	0.715
AF	1.61 (0.67–3.85)	0.286
Creatinine	1.00 (0.99–1.00)	0.399
BNP, per 100 pg/dl	1.43 (1.12–1.84)	0.005
LVEF (%)	0.96 (0.90–1.02)	0.143
LAVI (ml/m^2^)	0.98 (0.96–1.02)	0.355
E/e'	1.13 (1.07–1.20)	<0.001
PASP (mmHg)	1.03 (0.99–1.01)	0.084
TAPSE (mm)	0.94 (0.89–1.12)	0.952
S'(cm/s)	1.13 (0.99–1.30)	0.078
RVFAC (%)	0.92 (0.84–0.99)	0.038
RVFAC < 35%	3.54 (1.22–10.26)	0.02
RVEF (%)	0.73 (0.67–0.80)	<0.001
RVEF < 45%	3.73 (1.93–7.22)	<0.001
2D-RVFWLS (%)	1.12 (1.01–1.24)	0.035
2D-RVFWLS < 20%	2.88 (1.52–5.47)	0.001
3D-RVFWLS (%)	1.26 (1.14–1.41)	<0.001
3D-RVFWLS < 22%	4.60 (2.40–8.83)	<0.001

*2D, two dimensional; 3D, three dimensional; AF, atrial fibrillation; BNP, B-type natriuretic peptide; DBP, diastolic blood pressure; SBP, systolic blood pressure; NYHA, New York Heart Association; LVEF, left ventricular ejection fraction; LAVI, left atrial volume index; RVEF, right ventricular ejection fraction; RVFWLS, right ventricular free wall longitudinal strain; RVFAC, right ventricular fractional area change; TAPSE, tricuspid annular plane systolic excursion; PASP, pulmonary artery systolic pressure; AIC, Akaike information criterion; CI, confidence interval; HR, hazard ratio*.

**Table 3:** Multivariate analysis of predictors of poor outcomes in patients with heart failure and preserved ejection fraction.

	**Model 1**		**Model 2**		**Model 3**		**Model 4**	
**Variables**	**SBP** **+** **BNP** **+** **E/e**^****′****^**+** **FAC**		**SBP** **+** **BNP** **+** **E/e**^****′****^**+** **RVEF**		**SBP** **+** **BNP** **+** **E/e**^****′****^**+** **2D-RVFWLS**		**SBP** **+** **BNP** **+** **E/e**^****′****^**+** **3D-RVFWLS**	
	**HR (95% CI)**	***P***	**HR (95% CI)**	***P***	**HR (95% CI)**	***P***	**HR (95% CI)**	***P***
SBP, per 10 mmHg		0.210		0.399		0.793		0.234
BNP, per 100 pg/dl	1.39 (1.05–1.84)	0.020	1.55 (1.15–2.10)	0.005	1.45 (1.08–1.94)	0.013	1.56 (1.18–2.07)	0.002
E/e′	1.12 (1.06–1.19)	<0.001	1.07 (1.00–1.14)	0.41	1.10 (1.04–1.17)	0.002		0.065
RVFAC < 35%		0.461						
RVEF < 45%			3.47 (1.47–8.21)	0.005				
2D-RVFWLS < 20%					3.17 (1.54–6.53)	0.002		
3D-RVFWLS < 22%							5.73 (2.77–11.85)	<0.001
AIC	255		247		248		246	
C-index	0.69^*^		0.76^*^		0.74^*^		0.75^*^	

*2D, two dimensional; 3D, three dimensional; AF, atrial fibrillation; BNP, B-type natriuretic peptide; DBP, diastolic blood pressure; SBP, systolic blood pressure; NYHA, New York Heart Association; LVEF, left ventricular ejection fraction; LAVI, left atrial volume index; RVEF, right ventricular ejection fraction; RVFWLS, right ventricular free wall longitudinal strain; RVFAC, right ventricular fractional area change; TAPSE, tricuspid annular plane systolic excursion; PASP, pulmonary artery systolic pressure; AIC, Akaike information criterion; CI, confidence interval; HR, hazard ratio*.

* *The p-values < 0.05 were considered to indicate statistical significance*.

### Reproducibility

The intra-observer and inter-observer reproducibility are shown in Table 4. Intra-observer ICC was 0.83 for 2D-RVFWLS, 0.83 for 3D-RVFWLS, and 0.88 for RVEF. Inter-observer ICC was 0.83 for 2D-RVFWLS, 0.81 for 3D-RVFWLS, and 0.82 for RVEF. These parameters exhibited good reproducibility. Bland–Altman analysis demonstrated high agreement (intra-observer LOA: −4.87 to 5.26% for 2D-RVFWLS, −3.35 to 3.26% for 3D-RVFWLS, −5.99 to 5.87% for RVEF; inter-observer LOA: −5.43 to 3.82% for 2D-RVFWLS, −7.62 to 5.94% for 3D-RVFWLS, −6.37 to 7.77% for RVEF).

**Table 4:** Reproducibility of 2D- and 3D-STE parameters.

	**ICC**	**95% CI**	**Bias**	**LOA**
**Intra-observer**				
RVEF (%)	0.88	0.68–0.95	−0.06	−5.99 to 5.87
2D-RVFWLS (%)	0.83	0.58–0.94	0.20	−4.87 to 5.26
3D-RVFWLS (%)	0.83	0.57–0.94	−0.05	−3.35 to 3.26
**Inter-observer**				
RVEF (%)	0.82	0.57–0.94	0.70	−6.37 to 7.77
2D-RVFWLS (%)	0.83	0.59–0.94	−0.81	−5.43 to 3.82
3D-RVFWLS (%)	0.81	0.53–0.93	−0.90	−7.62 to 5.94

*2D, two dimensional; 3D, three dimensional; ICC, intraclass correlation coefficient; LOA, limits of agreement; RVEF, right ventricular ejection fraction; RVFWLS, right ventricular free wall longitudinal strain*.

## Discussion

To the best of our knowledge, this is the first study to comprehensively evaluate the prognostic value of RV function using 2D- and 3D-STE, and conventional echocardiographic indices in patients with HFpEF. The major findings of our study were (1) compared with HFpEF patients without end-point events, those with end-point events had lower RVEF and 3D-RVFWLS, whereas 2D-RVFWLS was not different. (2) 2D- and 3D-RVFWLS, and RVEF were independent predictors of poor outcomes in patients with HFpEF. (3) 3D-STE provided a similar predictive value as 2D-STE parameters in patients with HFpEF.

### RV Strain in HFpEF

In clinical practice, RV function assessment remains a challenge due to the complex shape and load dependency of the right ventricle. RV myocardial deformation imaging, which is less affected by geometric assumptions and loading conditions than traditional parameters, allows more sensitivity to detect the subclinical ventricular dysfunction in a variety of heart diseases (17). The previous study showed that RV longitudinal strain using 2D-STE was diminished in patients with HFpEF (18). However, 2D-STE is limited by 2D plane, foreshortened views, and out-of-plane motion of the speckles. The newly developed 3D-STE overcame the aforementioned limitations and could provide the comprehensive evaluation of RV performance. Currently, there are limited data regarding the RV function assessment using 3D STE, which mainly focus on patients with pulmonary hypertension (19–21). To our knowledge, this is the first study to evaluate RV performance using 3D-STE in patients with HFpEF.

Several mechanisms may be associated with RV dysfunction in patients with HFpEF, including increased pulmonary pressure, subtle LV dysfunction, neurohormonal interactions, and myocardial ischemia of the right ventricle (4, 22). Patients with HFpEF display an elevated LV filling pressure that leads to an increase in venous pulmonary pressures and hence in RV afterload. Indeed, our study corroborates the unfavorable impact of PASP on RV function, because the association of 2D- and 3D-STE parameters with PASP was noted in the present study. Additionally, although LVEF is preserved in HFpEF patients, there exists evidence of subtle LV systolic dysfunction (23). Ventricular interdependence could potentially affect RV strain measurement through the interaction of the interventricular septum.

### Prognostic Value of RV 2D- and 3D-STE Parameters

In recent years, there are growing data on the prognostic role of RV performance in patients with HF. Among them, RV 2D-STE analysis has been demonstrated to show the capability to quantify RV mechanics and provide prognostic information (10, 24–28). In a study of patients with HF with reduced ejection fraction (HFrEF), Motoki et al. reported that the RV 2D-STE strain offered an incremental value over LVEF and E/e′ ratio for predicting adverse clinical events (27). Likewise, Carluccio et al. also demonstrated that 2D-RVFWLS remained significantly associated with outcomes after further correction for LV systolic function parameters in patients with HFrEF (28). In another cohort of patients with HFrEF, Houard et al. demonstrated that the RV 2D-STE strain provided an additional value over CMR-RVEF, CMR-RVGLS, TAPSE, or FAC for predicting overall and cardiovascular mortality (11). In another observation study of patients with HFpEF, Lejeune et al. also showed that impaired RV global longitudinal strain provided significant additional prognostic value over clinical parameters in patients with HFpEF, whereas impaired TAPSE and RVFAC did not (24). However, 2D STE has inherent limitations. 3D STE theoretically circumvents the limitations of 2D STE; thus, a direct comparison between RV 2D- and 3D-STE parameters for predicting adverse clinical outcomes is clinically significant.

Till now, there are several studies regarding the prognostic value of RV 3D-STE indices in patients with pulmonary hypertension (12, 20, 21). Moreover, very few studies performed head-to-head comparisons of 2D-STE and 3D-STE in RV function evaluation. Previously, we revealed that 3D-RVFWLS is superior to 2D-RVFWLS in RV function assessment against CMR imaging in a study population with a wide variety of cardiovascular pathologies (29). Similarly, Nagata et al. demonstrated that LV 3D global LS is the most robust index for predicting adverse cardiac events in severe AS patients with preserved LVEF compared with 2D-STE parameters (30). To the best of our knowledge, this is the first study to estimate the prognostic significance of RV 3D-STE parameters in HFpEF patients and to directly compare its value with that of the 2D-STE strain. In our study, 2D- and 3D-RVFWLS, and RVEF were independently associated with poor clinical outcomes in patients with HFpEF. Moreover, the multivariate Cox hazard model revealed that 3D-RVFWLS and RVEF had a similar prognostic value as 2D-RVFWLS. Indeed, our study also revealed that patients with events displayed decreased RVEF and 3D-RVFWLS compared with event-free patients. In a large observational study of patients with various cardiovascular diseases, Nagata et al. (31) verified the incremental value of RVEF measured by 3D-STE over other echocardiography parameters including LV systolic and diastolic function for predicting adverse outcomes. However, they did not compare the prognostic value of 3D-RVFWLS and RVEF with that of 2D-STE parameters. We offer the first evidence that 3D-STE parameters were not superior to 2D-STE indices in patients with HFpEF for predicting future adverse outcomes. In summary, our study reinforces and expands the previous observations by demonstrating the usefulness of the RV strain in the risk stratification of patients with HFpEF. The present study not only confirms the prognostic significance of 2D-RVFWLS in patients with HFpEF but also indicates the similar prognostic value of 3D-STE as 2D-STE parameters.

### Clinical Implications

Considering that RV dysfunction is a risk factor of poor outcomes, RV function evaluation should be one part of the comprehensive assessment of patients with HFpEF. Although 3D STE has theoretical advantage over 2D-STE, experience of 3D-STE for RV evaluation is limited. Whether or not 3D-STE has superiority over 2D-STE requires clinical validation. Our study demonstrated that the 3D-STE strain is not superior to the 2D-STE strain in predicting adverse clinical outcomes in HFpEF patients. Consequently, our study revealed the important clinical implications because 2D-STE allows wider clinical application than 3D-STE in everyday clinical practice. However, the predictive value of 3D-STE in patients with HFpEF requires to be tested in future studies with a multicenter and large sample size.

### Limitations

Our study had some limitations. First, although patients in our study were consecutive, subjects with poor RV echocardiographic images quality were excluded from the analysis. This potentially limits the generalizability of our findings. Second, 3D-STE itself is limited by the low frame rates and suboptimal image quality, which may have effects on strain analysis and tracking quality. However, 3D-STE has been considered as clinically useful in LV function assessment. Third, we assessed only the RV longitudinal strain, rather than circumferential or radial deformation, because RV longitudinal strain plays a crucial role in the overall RVEF, and circumferential and radial strain could not be obtained using the current RV strain analysis software. Fourth, our findings pertain only to the software used in our study and may not extrapolate to other software algorithms because the STE measurements are hindered by the intervendor variability. Fifth, using 3D-STE indices to diagnose the severity of diseases in patients with HFpEF was not performed in our study. Future studies that investigate the diagnostic value of 3D-STE measurements in these patients could be the next step. Finally, this is a single-center study with a relatively limited sample size; future multicenter studies with larger sample sizes are required to determine the prognostic value of 3D-STE in patients with HFpEF.

## Conclusions

In summary, our study demonstrates that 2D- and 3D-RVFWLS, and RVEF can independently predict adverse clinical outcomes in patients with HFpEF. Moreover, 3D-RVFWLS and RVEF assessed by 3D-STE have similar predictive values as 2D-RVFWLS determined by 2D-STE. Therefore, these results of our study support the potential of RV 3D-STE to identify HFpEF patients at higher risk for adverse events.

## Take-Home Messages

3D-STE parameters were powerful predictors of poor outcomes, providing similar predictive values as 2D-STE indices in patients with HFpEF. These findings support the potential of RV 3D-STE to identify HFpEF patients at higher risk for adverse cardiac events.

## Data Availability Statement

The raw data supporting the conclusions of this article will be made available by the authors, without undue reservation.

## Ethics Statement

The studies involving human participants were reviewed and approved by Ethics Committee of Tongji Medical College, Huazhong University of Science and Technology. The patients/participants provided their written informed consent to participate in this study. Written informed consent was obtained from the individual(s) for the publication of any potentially identifiable images or data included in this article.

## Author Contributions

YLM, SSZ, YJX, WQW, JW, YLY, QL, MXX, and LZ: conception and design of the study. YLM, SSZ, YJX, YTZ, MZQ, LG, ML, and YXL: acquisition of data. YLM, SSZ, YJX, LZ, YML, and MXX: analysis and interpretation of data. LZ, YML, and MXX: drafting the article. All authors contributed to the article and approved the submitted version.

## Conflict of Interest

The authors declare that the research was conducted in the absence of any commercial or financial relationships that could be construed as a potential conflict of interest.

## Abbreviations

2D: two-dimensional
3D: three-dimensional
FAC: fractional area change
HFpEF: heart failure with preserved ejection fraction
HF: heart failure
HFrEF: heart failure with reduced ejection fraction
LVEF: left ventricular ejection fraction
RV: right ventricular
RVEDV: right ventricular end-diastolic volume
RVESV: right ventricular end-systolic volume
RVEF: right ventricular ejection fraction
RVFWLS: longitudinal strain of the right ventricular free wall
STE: speckle-tracking echocardiography
S′: tricuspid lateral annular systolic velocity
TAPSE: tricuspid annular plane systolic excursion.
